# Characterization of Antibody Interactions with the G Protein of Vesicular Stomatitis Virus Indiana Strain and Other Vesiculovirus G Proteins

**DOI:** 10.1128/JVI.00900-18

**Published:** 2018-11-12

**Authors:** Altar M. Munis, Maha Tijani, Mark Hassall, Giada Mattiuzzo, Mary K. Collins, Yasuhiro Takeuchi

**Affiliations:** a Division of Advanced Therapies, National Institute for Biological Standards and Control, South Mimms, United Kingdom; b Division of Infection and Immunity, University College London, London, United Kingdom; c Division of Virology, National Institute for Biological Standards and Control, South Mimms, United Kingdom; d Okinawa Institute of Science and Technology, Okinawa, Japan; University of Kentucky College of Medicine

**Keywords:** VSV, monoclonal antibodies, neutralizing antibodies, vesiculovirus

## Abstract

Vesicular stomatitis virus Indiana strain G protein (VSVind.G) is the most commonly used envelope glycoprotein to pseudotype lentiviral vectors (LV) for experimental and clinical applications. Recently, G proteins derived from other vesiculoviruses (VesG), for example, Cocal virus, have been proposed as alternative LV envelopes with possible advantages over VSVind.G. Well-characterized antibodies that recognize VesG will be useful for vesiculovirus research, development of G protein-containing advanced therapy medicinal products (ATMPs), and deployment of VSVind-based vaccine vectors. Here, we show that one commercially available monoclonal antibody, 8G5F11, binds to and neutralizes G proteins from three strains of VSV, as well as Cocal and Maraba viruses, whereas the other commercially available monoclonal anti-VSVind.G antibody, IE9F9, binds to and neutralizes only VSVind.G. Using a combination of G protein chimeras and site-directed mutations, we mapped the binding epitopes of IE9F9 and 8G5F11 on VSVind.G. IE9F9 binds close to the receptor binding site and competes with soluble low-density lipoprotein receptor (LDLR) for binding to VSVind.G, explaining its mechanism of neutralization. In contrast, 8G5F11 binds close to a region known to undergo conformational changes when the G protein moves to its postfusion structure, and we propose that 8G5F11 cross-neutralizes VesGs by inhibiting this.

**IMPORTANCE** VSVind.G is currently regarded as the gold-standard envelope glycoprotein to pseudotype lentiviral vectors. However, recently other G proteins derived from vesiculoviruses have been proposed as alternative envelopes. Here, we investigated two commercially available anti-VSVind.G monoclonal antibodies for their ability to cross-react with other vesiculovirus G proteins, identified the epitopes they recognize, and explored their neutralization activity. We have identified 8G5F11, for the first time, as a cross-neutralizing antibody against several vesiculovirus G proteins. Furthermore, we elucidated the two different neutralization mechanisms employed by these two monoclonal antibodies. Understanding how cross-neutralizing antibodies interact with other G proteins may be of interest in the context of host-pathogen interaction and coevolution, as well as providing the opportunity to modify the G proteins and improve G protein-containing medicinal products and vaccine vectors.

## Abstract (precis)

VSVind.G is currently regarded as the gold-standard envelope glycoprotein to pseudotype lentiviral vectors. However, recently other G proteins derived from vesiculoviruses have been proposed as alternative envelopes. Here, we investigated two commercially available anti-VSVind.G monoclonal antibodies for their ability to cross-react with other vesiculovirus G proteins, identified the epitopes they recognize, and explored their neutralization activity. We have identified 8G5F11, for the first time, as a cross-neutralizing antibody against several vesiculovirus G proteins. Furthermore, we elucidated the two different neutralization mechanisms employed by these two monoclonal antibodies. Understanding how cross-neutralizing antibodies interact with other G proteins may be of interest in the context of host-pathogen interaction and coevolution, as well as providing the opportunity to modify the G proteins and improve G protein-containing medicinal products and vaccine vectors.

## INTRODUCTION

The rhabdovirus vesicular stomatitis virus, Indiana strain (VSVind), has been used ubiquitously as a model system to study humoral and cellular immune responses, in addition to being a promising virus for oncolytic virotherapy against cancer (1–3). Furthermore, its single envelope G protein (VSVind.G) is the most commonly used envelope to pseudotype lentiviral vectors and serves as the gold standard in many experimental and clinical studies (4–6). Both receptor recognition and membrane fusion of the wild-type virus, as well as the pseudotyped particles, are mediated by this single transmembrane viral glycoprotein that homotrimerizes and protrudes from the viral surface (7–9). Recently, G proteins derived from other vesiculovirus subfamily members, namely, Cocal, Piry, and Chandipura viruses, have been proposed as alternative envelopes for lentiviral vector production due to some possible advantages over VSVind.G (10–12).

Although some antigenic and biochemical characteristics of VSVind.G have been reported (1, 7, 13–20), there is still little known about the other vesiculovirus G (VesG) proteins, and there is a general lack of reagents commercially available to identify, detect, and characterize them. In the past, monoclonal antibodies (MAbs) have been used to extensively study the antigenic determinants found on viral glycoproteins, e.g., hemagglutinin (HA) of influenza virus, the gp70 protein of murine leukemia virus (MLV), and rabies virus G protein (21–25). These previous studies, especially on the influenza virus strains and the rabies virus, have led to invaluable findings on the structure and function of the glycoproteins, allowing identification of epitopes essential in virus neutralization (25–27). In addition, MAbs have proven useful in viral pathogenesis studies, as mutants selected by antibodies in many cases demonstrated altered pathogenicity compared to that of their wild-type counterparts (28–30). Therefore, identification of antibodies that recognize VesG will not only be extremely valuable for vesiculovirus research but also aid in the development of G protein-containing advanced therapy medicinal products (ATMP) and vaccine vectors.

Here, we show two anti-VSVind.G antibodies, 8G5F11 and a goat polyclonal antibody, VSV-Poly (31, 32), can cross-react with a variety of the VesG proteins and cross-neutralize VesG-LV. We also demonstrate that the other commercially available extracellular monoclonal anti-VSVind.G antibody, IE9F9, lacks this cross-reactivity. We further characterize the two MAbs, 8G5F11 and IE9F9, with regard to their relative affinities toward various VesG proteins, binding epitopes, and cross-neutralization strengths.

(This article was submitted to an online preprint archive [33].)

## RESULTS

### Investigation of antibody cross-reactivity with VesG.

**FIG 1:**
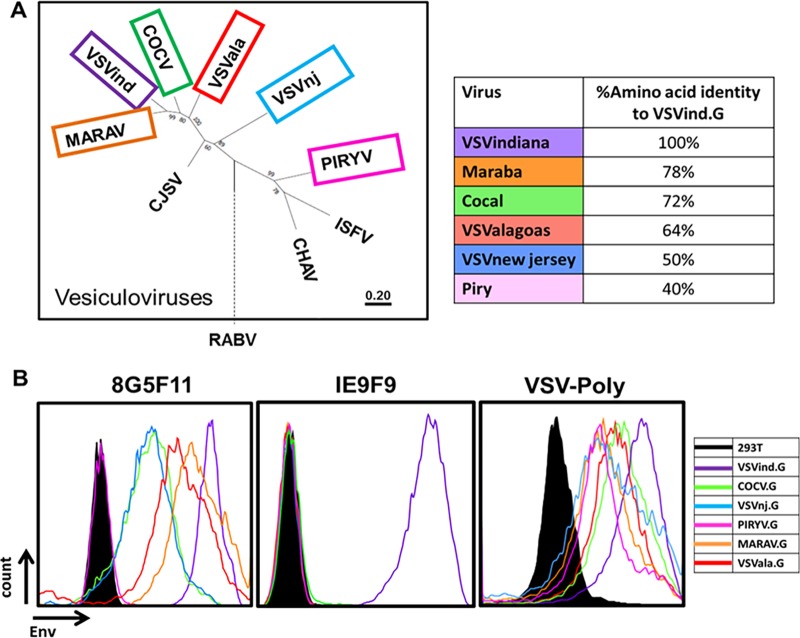
8G5F11 and VSV-Poly crossreact with a variety of VesG proteins, while IE9F9 only binds to VSVind.G. (A) G proteins of the major vesiculoviruses, as well as the G protein of the rabies virus (RABV), were analyzed with regard to their phylogenetic relationship. The tree of VesG proteins is drawn to scale, with branch lengths measured in the number of substitutions per site depicted in the linear scale. VSVind, vesicular stomatitis virus Indiana strain; COCV, Cocal virus; VSVnj, vesicular stomatitis virus New Jersey strain; PIRYV, Piry virus; CJSV, Carajas virus; CHAV, Chandipura virus; ISFV, Isfahan virus; MARAV, Maraba virus; VSVala, vesicular stomatitis virus Alagoas strain. Vesiculoviruses that we investigated are highlighted in boxes, and percent amino acid identities to VSVind.G are summarized in the table on the right. (B) Histograms represent the binding of the antibodies to the VesG expressed on the surface of transfected HEK293T cells. The strength of cross-reaction is depicted via the different MFIs of the histograms. Data shown are from one of the three repeats performed.

To investigate antibody binding to different vesiculovirus envelope glycoproteins (G proteins), we prepared plasmid pMD2-based vectors expressing six different vesiculovirus G (VesG) proteins: VSVind.G, Cocal virus G (COCV.G), vesicular stomatitis virus New Jersey strain G (VSVnj.G), Piry virus G (PIRYV.G), vesicular stomatitis virus Alagoas strain G (VSVala.G), and Maraba virus G (MARAV.G) (Fig. 1A). HEK293T cells were transfected with these plasmid constructs, stained with the different antibodies, and analyzed via flow cytometry. While IE9F9 only bound to VSVind.G, anti-VSVind.G monoclonal antibody 8G5F11 and VSV-Poly both could recognize various VesG proteins with various binding strengths (Fig. 1B). PIRYV.G, the most distant vesiculovirus G investigated, with approximately 40% identity to VSVind.G on the amino acid level, could be recognized by VSV-Poly, while 8G5F11 did not bind to it.

### Characterization of IE9F9 binding, 8G5F11 cross-reactivity, and its affinity toward other VesGs.

**FIG 2:**
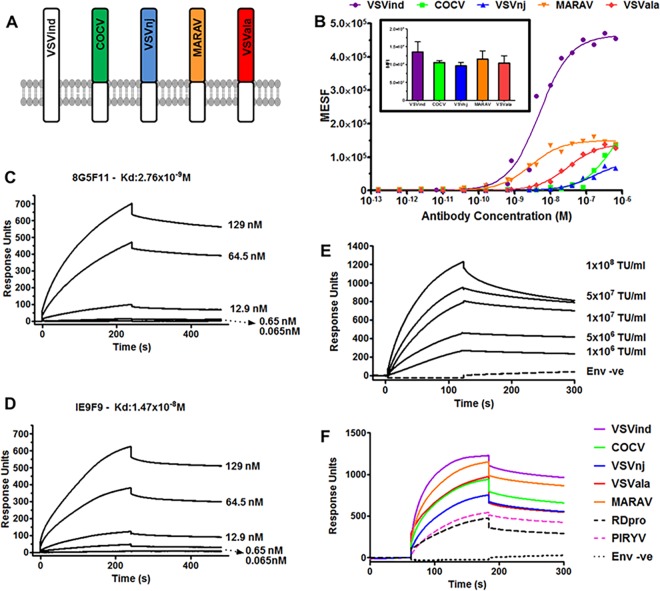
Investigation of 8G5F11 and IE9F9 affinities toward VSVind.G and characterization of 8G5F11 cross-reactivity. (A) Schematic representation of the chimeric vesiculovirus G proteins with VSVind.G transmembrane and C-terminal domains. (B) HEK293T cells expressing chimeric VesG were incubated with serial dilutions of 8G5F11 and analyzed via flow cytometry. MFIs of the fluorescent signals were converted into the number of fluorophores using the MESF standard curve according to the manufacturer’s instructions, the background signal from mock-transfected HEK293T cells was subtracted, and binding saturation curves were plotted. The various affinities of the mAb toward different VesG is demonstrated by the shift in the slope of the binding curves. The curves were fitted and dissociation constants (*K_d_*) were calculated using the software GraphPad Prism 5, modeling the interaction as 1:1 specific binding: VSVind.G, 2.64 × 10^−9^ M; COCV.G, 5.88 × 10^−7^ M; VSVnj.G, 1.57 × 10^−7^ M; MARAV.G, 4.13 × 10^−9^ M; VSVala.G, 3.09 × 10^−9^ M. Data shown represent the mean of three repeats performed in duplicates. (Inset) The expression levels of the chimeric G proteins were determined via intracellular P5D4 staining. Data shown represent the means ± SD from three repeats performed in duplicates. Surface plasmon resonance (SPR) analysis of 8G5F11 (C) and IE9F9 (D) binding to immobilized Gth in HBS-EP buffer. (E) Surface plasmon resonance analysis of VSVind.G-LV binding to immobilized 8G5F11 in HBS-EP buffer. (F) Surface plasmon resonance analysis of Ves.G-LV (1 × 10^8^ TU/ml) binding to immobilized 8G5F11 in HBS-EP buffer. The binding curves are normalized with regard to the relative response of unenveloped LV particles (env -ve), which is regarded as the background. SPR data shown are from one of the three repeats performed.

To confirm that the difference in 8G5F11 binding to VesG was indicative of the MAb affinity toward VesG and not a difference in relative expression levels of the G proteins, we synthesized chimeric G proteins. The endogenous transmembrane and C-terminal domains of VesG were switched with that of VSVind.G (Fig. 2A). Following the expression of these chimeric G proteins in HEK293T cells, we investigated 8G5F11 and IE9F9 binding saturation using quantitative flow cytometry, while the relative expression levels of the G proteins were monitored using an intracellular anti-VSVind.G MAb, P5D4 (Fig. 2B). 8G5F11 showed a wide range of affinities toward VesG. While its affinity for MARAV.G was comparable to that of VSVind.G, its interactions with COCV.G and VSVnj.G were much weaker.

To consolidate this finding, we further investigated these MAb-G protein interactions via surface plasmon resonance (SPR). First, to quantify MAb binding to G protein monomers under conformationally correct folding, we immobilized wild-type (wt) VSVind.G produced by thermolysin-limited proteolysis of viral particles (Gth) (7, 17) and tested the dose-dependent binding of the two MAbs (Fig. 2C and D). The measured dissociation constant (*K_d_*) values for 8G5F11 and IE9F9 binding to VSVind.G were 2.76nM and 14.7nM, respectively. To further analyze the VesG-8G5F11 interaction, we immobilized the MAb and investigated VesG-pseudotyped lentiviral vector (LV) binding. Since pseudotyped LV particles contain many trimeric G protein spikes (34), the analysis of the interaction between VesG binding to immobilized 8G5F11 reflects avidity. A specific, vector dose-dependent binding (i.e., increasing binding response with increasing titers) of VSVind.G was detected which saturated faster than the MAb-Gth interaction (Fig. 2E). When identical doses of VesG-LV at 1 × 10^8^ transduction units (TU)/ml were injected on immobilized 8G5F11, we observed patterns of binding similar to those seen with quantitative flow cytometry, in the order of strength of VSVind > MARAV > VSVala > Cocal > VSVnj (Fig. 2F). Unrelated RDpro envelope pseudotyped LVs were utilized as a negative control to deduce unspecific interaction of enveloped particles with immobilized MAb. PIRYV.G-LV demonstrated a response similar to that of RDpro-LV, indicative of the lack of binding between the G protein and 8G5F11.

### Determining the cross-neutralization abilities of anti-VSVind.G antibodies.

**FIG 3:**
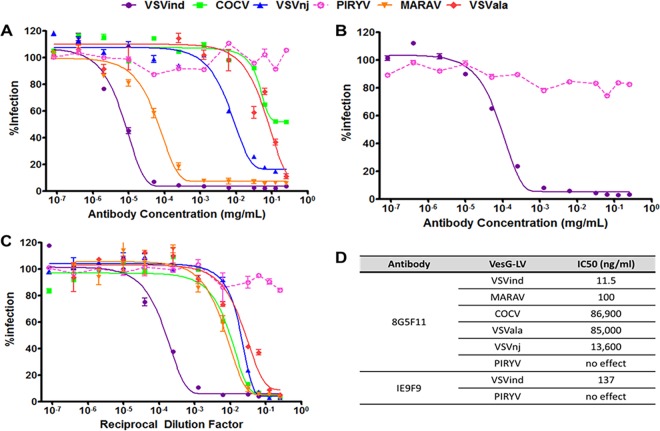
Neutralization activity of MAbs and VSV-Poly. Neutralization of VesG-LV by 8G5F11 (A), IE9F9 (B), and VSV-Poly (C). Solid lines signify the neutralization effect observed, while the dotted lines indicate the lack of neutralization. (D) Calculated IC_50_ values for 8G5F11 and IE9F9, depicting the potency of neutralization. The curves were fitted using the software GraphPad Prism 5 and modeled as an inhibitor versus response curve with variable Hill slopes and IC_50_ values calculated. Data shown represent the means ± SD from three repeats.

These three antibodies were evaluated for their ability to neutralize VSVind.G and VesG pseudotyped LVs (Fig. 3). 8G5F11 demonstrated various strengths of neutralization against VesG-pseudotyped LVs, with IC_50_ values ranging from 11.5 ng/ml to 86.9 µg/ml (Fig. 3A). There was, however, limited correlation between G proteins’ binding strength and sensitivity of LV, e.g., VSVnj.G-LV was more sensitive than COCV.G-LV (Fig. 3A), while COCV.G binding was stronger (Fig. 1 and 2). IE9F9 neutralized only VSVind.G-LV at an IC_50_ of 137 ng/ml, about 12-fold weaker than 8G5F11 (Fig. 3B). In the case of VSV-Poly, we observed cross-neutralization only at high serum concentrations (Fig. 3C). Furthermore, although VSV-Poly bound to PIRYV.G, it did not neutralize PIRYV.G-LVs.

### Mapping the epitopes of anti-VSVind.G MAbs and identification of key amino acid residues that dictate antibody binding and neutralization.

**FIG 4:**
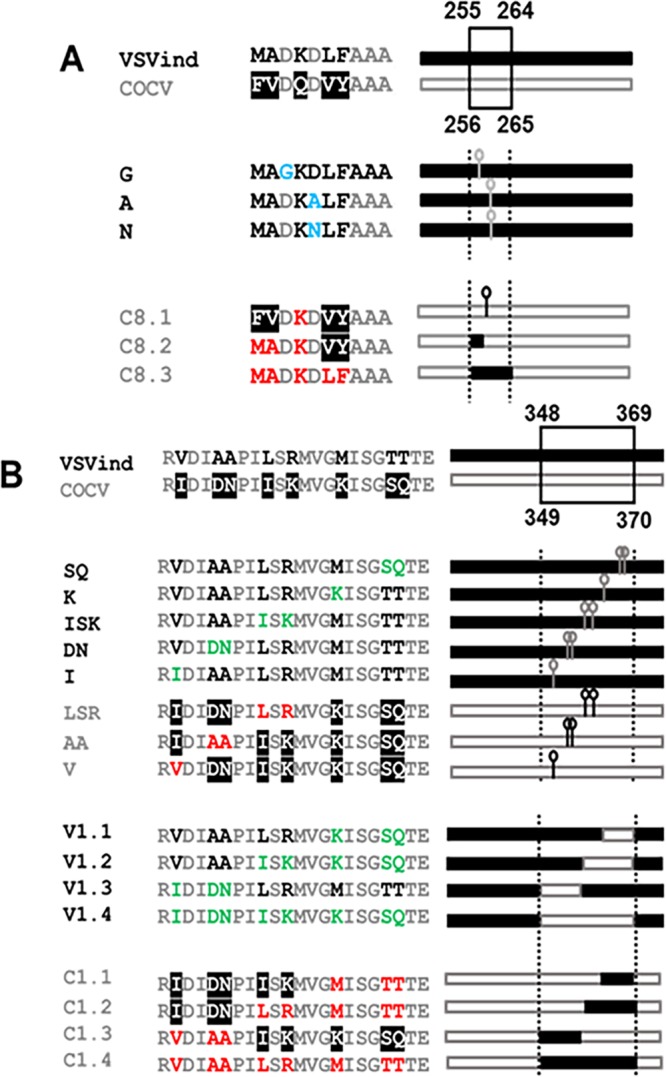
Mutants and chimeric G proteins produced for epitope mapping. Mutants and chimeras produced for epitope mapping of monoclonal antibodies 8G5F11 (A) and IE9F9 (B). Names and linear representations of the mutants and chimeras are listed on either side of the amino acid alignments of the regions where mutations were made. The boundaries are labeled with respective amino acid numbers. Amino acid alignment legend: black, residues from wt VSVind.G; white with black background, residues from wt COCV.G; gray, shared residues; blue, previously identified mutants (15); red, VSVind.G residues switched into COCV.G; green, COCV.G residues switched into VSVind.G. For linear G protein representations, the regions were the mutations were carried out are represented by dotted lines. Black bars represent wt VSVind.G sequences, while gray-bordered bars are for wt COCV.G residues. Point mutations are denoted by a bar and a circle.

To map where the neutralizing antibodies might bind on the G protein surface, a series of chimeric G proteins between VSVind.G and COCV.G were constructed. The initial binding and neutralization studies performed with these chimeras enabled us to specify the location of the epitopes of these MAbs to between amino acid residues 137 and 369 on VSVind.G (data not shown). Furthermore, looking at previously published data on 8G5F11’s and IE9F9’s epitopes obtained through mutant virus escape assays (1, 13–15), we concentrated on two distinct regions on VSVind.G and synthesized 22 different mutant G proteins to study the epitopes (Fig. 4). The mutants were cloned into the pMD2 backbone and their functionality investigated via LV infection and antibody binding assays. All G proteins were confirmed to be functional and could successfully pseudotype LVs, yielding titers comparable to those of their wt counterparts. Furthermore, their relative expression levels were monitored by intracellular P5D4, which also recognizes the intracellular domain of COCV.G. Lastly, they could be detected by extracellular VSV-Poly, implying there were not any substantial protein display issues (data not shown).

**FIG 5:**
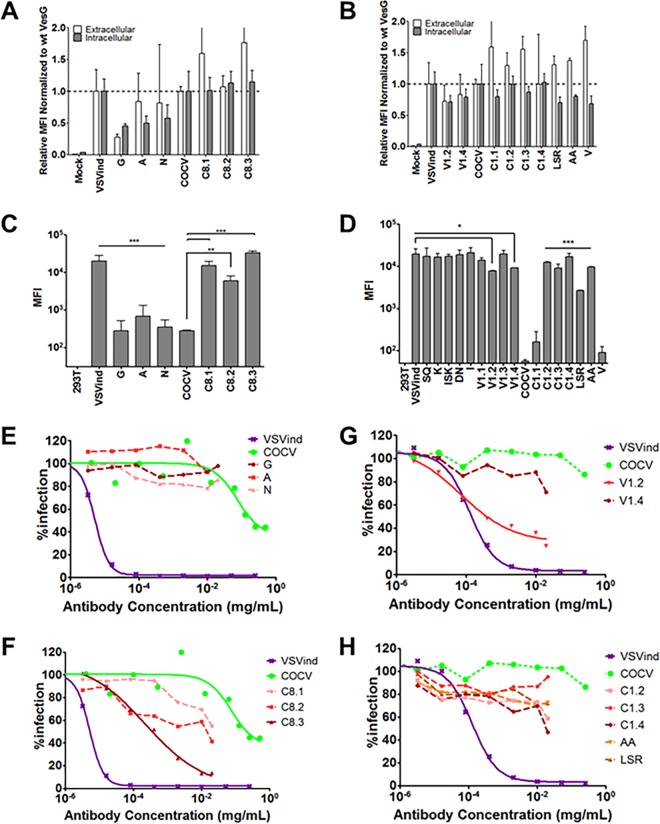
Investigation of antibody binding to mutant G proteins and neutralization of mutant LVs. HEK293T cells were transfected to express the mutant G proteins on their surface. (A and B) The cells expressing chimeric mutants were stained with extracellular VSV-Poly (white bars) and intracellular P5D4 (gray bars) as an expression control for the G proteins. The measured MFI values were normalized to the wt VesG signals for each set of mutants. The same population of cells was also incubated with 8G5F11 (C) and IE9F9 (D) at saturating concentrations. One-way analysis of variance with Dunnett’s posttest was performed to compare the MFI values of mutant G proteins to that of their wild-type counterpart. Lines with right angles above the bars denote the significance of a single comparison, while straight lines signify that all of the individual comparisons within the group share the denoted significance unless otherwise stated (*, *P*  < 0.05; **, *P*  < 0.01; ***, *P*  < 0.001). This assay was performed three times in duplicates; means ± SD are plotted above. The neutralization curves for select mutant and chimeric G-pseudotyped LVs are plotted for 8G5F11 (E and F) and IE9F9 (G and H). Solid lines signify the neutralization effect observed. (E and G) Previously reported reductions in binding for VSVind.G mutants translated into either complete or partial resistance to neutralization by both antibodies. For COCV.G mutants (F and H), the mutations conferred the G protein sensitivity to neutralization by 8G5F11 but not by IE9F9. The curves were fitted using the software GraphPad Prism 5, modeled as an inhibitor versus response curve with variable Hill slopes. Data shown represent the means from three experiments performed in independent triplicates.

We first investigated antibody binding to these G proteins via flow cytometry. Extracellular VSV-Poly and intracellular P5D4 stains determined relative expression levels of the mutants. For both sets the relative difference between expression levels of mutant and wt proteins was less than 2-fold in most cases (Fig. 5A and B). In the case of 8G5F11, binding to VSVind.G mutants was reduced by approximately 100-fold, while the changes on COCV.G enabled these mutants to bind to 8G5F11 at levels similar to that of wt VSVind.G (Fig. 5C). This change in binding could also be observed on a Western blot: while none of the VSVind.G mutants could be visualized, 8G5F11 could bind to the COCV.G chimera C8.3 (data not shown). It can be inferred from these results that amino acids 257 to 259 (DKD) are the key residues that dictate 8G5F11 binding to G proteins.

On the other hand, for IE9F9 no statistically significant changes in antibody binding were observed for VSVind.G mutants (data not shown), except for chimeras V1.2 and V1.4 (Fig. 5D). However, there was a substantial gain of binding effect for COCV.G mutants. While IE9F9 does not bind to wt COCV.G, mutations of amino acid residues LSR and AA (Fig. 4) alone led to a significant increase in the fluorescence signal, and thereby antibody binding. C1.4 with both LSR and AA had a median fluorescence intensity (MFI) level comparable to that of wt VSVind.G.

The neutralization profile of both VSVind.G and COCV.G mutants was also examined (Fig. 5E to H). While LVs pseudotyped with VSVind.G mutants G, A, and N were not neutralized by 8G5F11 (Fig. 5E), various degrees of sensitivity were observed for COCV.G mutants, with the strongest binder being the most sensitive (Fig. 5F). On the other hand, this was not the case for IE9F9 mutants. While dose-dependent neutralization of V1.2-LV was observed, VSVind.G mutant V1.4-LV was resistant to IE9F9 neutralization (Fig. 5G). Furthermore, no effect was observed on COCV.G mutant LV infection even though all bound to the MAb, some at levels similar to those of wt VSVind.G (Fig. 5H). The data show that while 8G5F11 employs a neutralization mechanism that is effective among the tested VesG, IE9F9’s is VSVind.G specific and binding does not necessarily result in neutralization.

### Investigation of neutralization mechanisms utilized by the MAbs: binding competition with LDLR.

**FIG 6:**
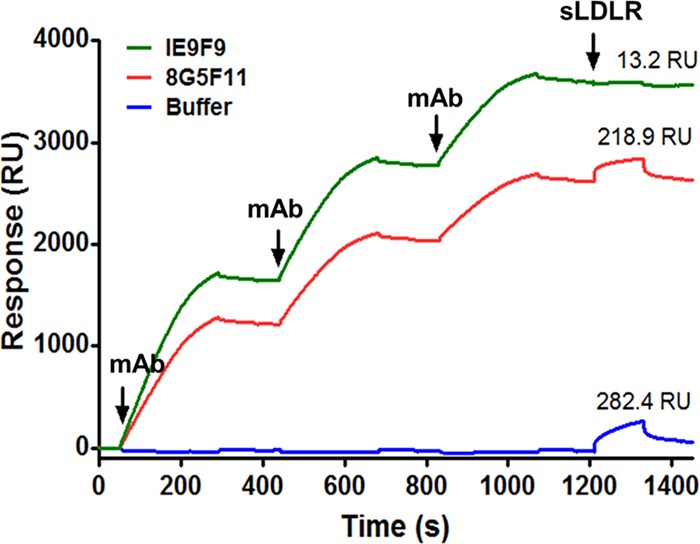
IE9F9 hinders sLDLR binding to Gth. 8G5F11 and IE9F9 were injected over immobilized Gth at a 10 µg/ml concentration three times to achieve binding saturation. Following this, sLDLR was injected over the chip at a concentration of 10 µg/ml and its binding to Gth was measured. As a buffer control, an identical sLDLR injection was performed following multiple injections of HBS-EP running buffer. Measured sLDLR binding levels are indicated above the binding response curves, and times of injections are marked with arrows. The data presented represent one of the three repeats performed.

Antibodies neutralize viruses and viral vectors by several mechanisms. Many neutralizing antibodies (NAbs) prevent virions from interacting with cellular receptors (35). VSVind.G’s major receptor has been identified as the low-density lipoprotein receptor (LDLR) (34, 36). Therefore, we investigated the binding competition between 8G5F11 and IE9F9 with LDLR via SPR as a potential neutralization mechanism for the MAbs (Fig. 6). Gth immobilized on the chip surface was saturated with repeated injections of 8G5F11 and IE9F9. This was followed by an injection of recombinant soluble human LDLR (sLDLR), and its binding to Gth was examined. While sLDLR was able to bind to Gth following 8G5F11 saturation as well as Gth without antibody exposure (buffer control), this interaction was almost completely abrogated by IE9F9. These data suggest that IE9F9, but not 8G5F11, neutralizes VSVind.G-LV by blocking the G protein-receptor interaction either through steric hindrance or direct competition.

### 8G5F11 blocks infection after endocytosis and before genome reverse transcription.

**FIG 7:**
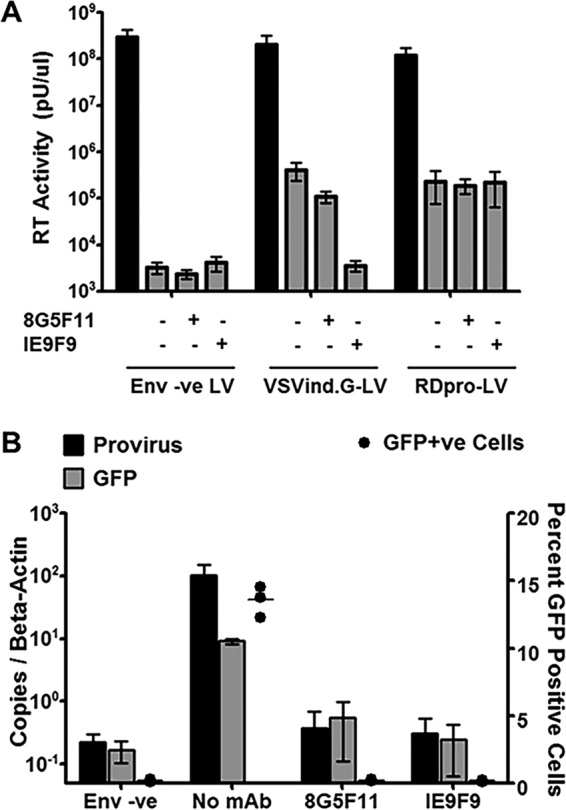
Internalization but not reverse transcription of 8G5F11 bound LVs. (A) VSVind.G- and RDpro-LVs as well as env -ve LVs were incubated with plain Opti-MEM, 8G5F11, or IE9F9 and plated on HEK293T cells. After allowing internalization of the particles, cells were lysed and RT activity measured via SG-PERT. The black bars represent the initial viral inputs plated on cells. The data shown represent means ± SEM from two repeats performed in triplicate. (B) In parallel infections total DNA was extracted 5 h postinfection, and reverse-transcribed provirus and transgene copies were quantified via qPCR and normalized to β-actin copies. The data shown represent means ± SEM from an experiment performed in independent triplicates. GFP expression was determined 48 h postinfection via flow cytometry. Each point represents an independent triplicate, and the line stands for the median.

As demonstrated by the SPR data, 8G5F11 did not block receptor binding of the G protein, implying that it is acting on LV infection steps following receptor binding. Therefore, we investigated the internalization of 8G5F11-bound LV particles (Fig. 7A). For this, VSVind.G- and RDpro-LV, as well as unenveloped (env -ve) LVs, were incubated with MAbs or plain Opti-MEM and plated on HEK293T cells. The level of LV which was internalized and therefore resistant to cell trypsinization was measured through reverse transcriptase (RT) activity 30 min postinfection. RT activity measured in env -ve samples were regarded as unspecific uptake and as background. RDpro-LVs, regardless of incubation with anti-VSVind.G MAbs, were internalized, and so were VSVind.G-LVs in Opti-MEM. While VSVind.G-LV incubated with IE9F9 demonstrated RT activity levels comparable to that of unenveloped LVs, 8G5F11-bound LV particles were endocytosed, displaying RT activity similar to that of Opti-MEM-mixed VSVind.G-LV. In parallel infections, total DNA was harvested 5 h postinfection from VSVind.G-LV-infected samples to determine reverse-transcribed provirus and transgene (GFP) copies via quantitative PCR, and GFP expression was determined 48 h postinfection via flow cytometry (Fig. 7B). Reverse-transcribed LV copies and GFP expression were only detected in no-MAb infections. Taken together, the data suggest that 8G5F11 blocks VSVind.G-LV infection following receptor binding and endocytosis of the vectors and before genome reverse transcription.

## DISCUSSION

VSVind.G is the most commonly used envelope glycoprotein to pseudotype LVs for experimental and clinical applications. VSVind.G-pseudotyped LVs can be produced at high titers and can infect a range of target cells. However, VSVind.G is cytotoxic to cells; thus, it is difficult to express it constitutively (37, 38). Moreover, VSVind.G-pseudotyped LVs can be inactivated by human serum complement, which limits their potential *in vivo* use (39–43). Therefore, there is a clear need for alternative envelopes to pseudotype LVs. Some of the most recent alternative envelopes that have been utilized are the G proteins of the other vesiculovirus family members (10–12). However, one drawback of using these new G proteins is that there are no reagents commercially available to identify or characterize them.

In this study, we report that the commercially available anti-VSVind.G monoclonal antibody 8G5F11 can, unlike VSVind.G-specific IE9F9, cross-react with a variety of the VesG proteins and cross-neutralize VesG-LV. Furthermore, we explored the functional epitopes for both MAbs, identifying new amino acid substitutions in addition to previously reported ones (15), and elucidated their mechanism of neutralization. G proteins of vesiculoviruses other than VSVind are being utilized for LV pseudotyping with the construction of COCV.G-LV producer clones for *gfp* and T cell receptor-encoding LVs, and the use of PIRYV.G and CHAV.G in transient LV production has been reported (10, 12, 44). We believe that the work presented will lay the groundwork for adaptation of VesG into new G-protein-based ATMP and allow for the utilization of these commercially available antibodies in vesiculovirus and VesG-based gene therapy research.

The cross-reactive monoclonal 8G5F11 demonstrated interesting characteristics. Its high cross-reactivity, even toward more distant relatives of VSVind.G, such as VSVnj.G, suggested that it recognizes a well-conserved epitope. However, the results of the binding saturation assay did not correlate with phylogenetic relativity. It revealed that its affinity toward COCV.G, one of the closest relatives of VSVind.G, was one of the weakest among the VesG proteins investigated, with an almost 250-fold difference compared to VSVind.G (Fig. 2B).

**FIG 8:**
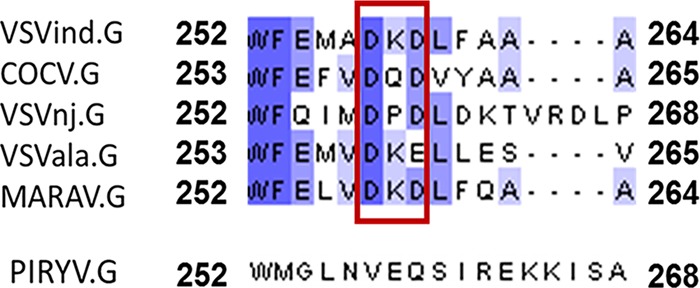
Comparison of 8G5F11’s epitope in other VesG through amino acid alignment. Amino acid residues for the vesiculovirus G proteins were retrieved from UniProt. The sequences were aligned using the ClustalOmega online multiple sequence alignment tool (EMBL-EPI), and the alignments were visualized using JalView software (53). The boundaries are labeled with respective amino acid numbers. Dashed lines represent gaps introduced to maximize matching of amino acid residues. Blue shading indicates percent identity: dark, 80% to 100%; medium, 60% to 80%; light, 40% to 60%; no color, <40% identity. Amino acid residues that dictate 8G5F11 binding are highlighted in a red box.

This discrepancy can be explained through fine mapping of the 8G5F11 epitope. We identified the amino acids 257 to 259, DKD, as the key residues on VSVind.G for 8G5F11 binding. On VSVind.G the two negatively charged aspartic acid residues flank the positively charged lysine, possibly contributing to the structure of the α-helix form through salt bridges (7, 16, 17). When either of the aspartic acid residues is mutated to a neutral residue, a significant reduction in binding is observed. When this is compared to the corresponding three residues on other VesG proteins, the antibody binding is dependent on the overall charge of these three residues rather than the ones surrounding them. In MARAV.G, these residues are identical to those of VSVind.G, explaining why the antibody has strength of binding similar to that of these two G proteins (Fig. 8). On the other hand, VSVala.G binds 8G5F11 with high affinity, although these residues are not fully conserved, as in VSVala.G the second aspartic acid residue is replaced with a glutamic acid. However, it is possible that the conservation of the second negative charge and the structural similarities between these two residues enable a robust G protein-antibody interaction. Lastly, the corresponding amino acid residues in PIRYV.G, VEQ, have electrostatically and structurally different characteristics from those of lysine and aspartic acid, leading to the lack of interaction between the MAb and G protein.

We showed that IE9F9 recognizes a β-sheet-rich domain of the G protein (7, 17). A complete abrogation of binding was not observed with the VSVind.G mutants produced. This implies that the antibody either relies on other structural cues and environmental charges around for binding or can utilize a secondary epitope. However, through the gain of binding effect observed in COCV.G mutants, we were able to identify two regions, AA and LSR, amino acid residues 352 to 353 and 356 to 358, respectively, on VSVind.G that are the key to this antibody’s interaction.

All three reagents investigated demonstrated neutralizing activities. 8G5F11 had the greatest ability to cross-neutralize a wide array of vesiculovirus family members. The strength of neutralization for this MAb, however, did not correlate with its affinity toward other VesG proteins (Fig. 2 and 3). This suggests that innate differences, such as protein structure, between the VesG proteins plays a role in LV neutralization. Since the structures of the VesG other than VSVind.G and CHAV.G are not yet delineated, it is hard to point out the key factors and mechanism involved accurately. However, we have identified 8G5F11’s epitope as lying close to the crossover point between pleckstrin homology and the trimerization domain of VSVind.G (7, 17, 19, 20, 36). Several hinge segments have been identified in the proximity of the epitope that undergo large rearrangements in relative orientation, while the G protein refolds from pre- to postfusion conformation under the low-pH conditions of the endosomes following endocytosis (16, 19, 36). It can be hypothesized that 8G5F11 hinders this process, ultimately preventing viral fusion and infection. As pH-induced conformational change during viral fusion is a shared characteristic among VesG (45), this might be the underlying reason behind 8G5F11’s ability to cross-neutralize VesG-LV.

We have shown that IE9F9 blocks VSVind.G binding to its major receptor, LDLR (Fig. 6). The crystal structures of VSVind.G in complex with LDLR domains have been recently identified and have shown that VSVind.G can interact with two distinct cysteine-rich domains (CR2 and CR3) of LDLR (36). One of the regions on VSVind.G that is crucial for LDLR CR domain binding lies between amino acids 366 and 370, only seven amino acids away from the key residues in IE9F9’s epitope. The key residues in this region of VSVind.G are not conserved among vesiculoviruses; therefore, the use of neither this epitope nor LDLR can be generalized to the other members of the genus, making IE9F9’s epitope and neutralization mechanism specific to VSVind.G. The lack of cross-reactivity and cross-neutralization (Fig. 1 and 3) displayed by the MAb toward VesG, as well as its failure to neutralize COCV.G mutants when its epitope is inserted into the G protein (Fig. 5), suggest a specific requirement for binding mode between IE9G9 and G proteins to result in neutralization. Nikolic and colleagues have demonstrated that VSVind.G has specifically evolved to interact with the CR domains of other LDLR family members (36). The other members of the receptor family have already been identified as secondary ports of entry for the virus (34). Complete neutralization achieved with IE9F9 indicates that the other LDLR family members are interacting with the same epitope on VSVind.G as well.

On the other hand, 8G5F11 does not interfere with receptor recognition (Fig. 6) and allows internalization of the LV particles by the target cells (Fig. 7A). However, the vector genome does not get reverse transcribed and infection does not occur, implying 8G5F11 interferes with infection mechanisms after receptor binding and internalization of the particles. As discussed above, 8G5F11’s epitope is located at the PH domain of the G protein in an α-helix around hinge regions that undergo structural rearrangement. Our results, therefore, suggest that 8G5F11 neutralizes VesG by interfering with such conformational changes and membrane fusion.

Further work on these two identified epitopes regarding their immunodominance in an *in vivo* setting and their detailed characterization on other VesG proteins from a structure-function point of view may be of interest in the context of host-pathogen interaction and coevolution. This may also provide the opportunity for modifying VSVind.G to improve G protein-containing ATMP and VSVind-based vaccine vectors.

## MATERIALS AND METHODS

### Cell culture.

In all experiments, HEK293T cells were used. The cell line was maintained in Dulbecco's modified Eagle’s medium (DMEM) (Sigma-Aldrich, St. Louis, MO) supplemented with 10% heat-inactivated fetal calf serum (Gibco, Carlsbad, CA), 2 mM l-glutamine (Gibco), 50 U/ml penicillin (Gibco), and 50 µg/ml streptomycin (Gibco). All cells were kept in cell culture incubators at 37°C and 5% CO_2_.

### Phylogenetic analysis of vesiculovirus and rabies virus G proteins based on amino acid sequences.

G proteins of the major vesiculoviruses (VSVind, UniProt accession number P03522; Cocal virus, O56677; VSVnj, P04882; Piry virus, Q85213; Maraba virus, F8SPF4; VSVala, B3FRL4; Chandipura virus, P13180; Carajas virus, A0A0D3R1Y6; Isfahan virus, Q5K2K4), as well as the G protein of the rabies virus (Q8JXF6), were included in the analysis. The amino acid sequences were aligned using the ClustalOmega online multiple-sequence alignment tool (EMBL-EPI). The evolutionary analyses were conducted in MEGA7 (46). The evolutionary history was inferred by using the maximum likelihood method based on the Jones-Taylor-Thornton matrix-based model (47). The tree with the highest likelihood is shown with the bootstrap confidence values (out of 100) indicated at the nodes. The tree is drawn to scale, with branch lengths measured in the number of substitutions per site, depicted in the linear scale. It should be noted that the amino acid sequence of the full-length G proteins (including the signal peptide) were referred to in the manuscript. Accordingly, reference to specific residue numbers is made in the context of these full-length sequences.

### Plasmids used in experiments.

VSVind.G expression plasmids, pMD2.G, and gag-pol expression plasmid p8.91 (48) were purchased from Plasmid Factory (Germany). GFP expressing self-inactivating vector plasmid used in the production of lentiviral vectors was produced in our laboratory previously (50). pMD2.Cocal.G, a COCV.G expression plasmid, was kindly provided by Hans-Peter Kiem (Fred Hutchinson Cancer Research Center, Seattle, WA). All other VesG envelopes were cloned into this backbone using the restriction enzymes PmlI and EcoRI. Amino acid sequences for VSVnj.G, PIRYV.G, MARAV.G, and VSVala.G were retrieved from UniProt. Codon-optimized genes were ordered from Genewiz (South Plainfield, NJ). Unrelated feline endogenous virus RD114-derived RDpro envelope (50) was used as a negative control.

### Gene transfer to mammalian cells.

Single plasmid transfection was used to express VesG on HEK293T cell surfaces. HEK293T cells were seeded on the day prior to transfection at 4 × 10^6^ cell per 10-cm plate. These cells were transfected by lipofection using FuGENE6 (Promega, Madison, WI) according to the manufacturer’s instructions. The cells were harvested 48 h later to be used in various flow cytometry assays.

### Overlapping extension PCR to synthesize VesG chimeras.

A Phusion high-fidelity PCR kit (NEB, Ipswich, MA) was used to perform the PCRs. All primers used were obtained from Sigma-Aldrich. To splice two DNA molecules, special primers were at the joining ends. For each molecule, the first of two PCRs created a linear insert with a 5′ overhang complementary to the 3′ end of the sequence from the other gene. Following annealing, these extensions allowed the strands of the PCR product to act as a pair of oversized primers and the two sequences were fused. Once both DNA molecules were extended, a second PCR was carried out with only the flanking primers to amplify the newly created double-stranded DNA of the chimeric gene.

### Surface plasmon resonance.

SPR analyses were performed using a BIAcore T100 instrument (GE Healthcare). Gth (0.04 mg/ml) and 8G5F11 (0.03 mg/ml) in sodium acetate buffers (10 mM, pH 4.5 and 4.0, respectively) were immobilized on a CM5 sensor chip using the amine coupling system according to the manufacturer’s instructions. To measure MAb affinity to VSVind.G, 8G5F11 (molecular weight [MW], 155 kDa) and IE9F9 (MW, 155 kDa) were suspended in HBS-EP (0.01 M HEPES, pH 7.4, 0.15 M NaCl, 3 mM EDTA, 0.005, vol/vol, P20) and passed over the immobilized Gth at the indicated concentrations. To measure VesG-LV avidity against 8G5F11, LV preparations were suspended in HBS-EP buffer and passed over the immobilized MAb at the indicated titers. The dissociation constants were calculated using BIAevaluation software according to the manufacturer’s instructions. For the competitive binding assay, multiple injections of MAbs at 10 µg/ml concentration were performed, followed by injection of soluble recombinant LDLR (R&D Systems, Minneapolis, MN) at an identical concentration.

### Use of MESF system for quantitative flow cytometry analysis.

Quantum Alexa Fluor 647 molecules of equivalent soluble fluorochrome (MESF) kit (Bangs Laboratories, Fishers, IN) was utilized for all quantitative fluorescence flow cytometry experiments. This is a microsphere kit that enables the standardization of fluorescence intensity units. Beads with a predetermined number of fluorophores are run on the same day and at the same fluorescence settings as stained cell samples to establish a calibration curve that relates the instrument channel values (i.e., median fluorescence intensity [MFI]) to standardized MESF units.

### Extracellular and intracellular antibody binding assay.

HEK293T cells were transfected to express the G proteins. Forty-eight h later cells were harvested, washed twice with phosphate-buffered saline (PBS), and plated in U-bottom 96-well plates at identical densities. For intracellular antibody binding assays, cells were fixed with 1% formaldehyde (Sigma-Aldrich, St. Louis, MO) in PBS, permeabilized using 0.05% saponin (Sigma-Aldrich, St. Louis MO) in PBS, and blocked with 1% bovine serum albumin (BSA; Sigma-Aldrich, St. Louis MO) in PBS. Cells were then incubated with serial dilutions of extracellular and intracellular antibodies, ranging from 0.1 mg/ml to 2 × 10^−7^ mg/ml in 1% BSA (Sigma) in PBS in a total reaction volume of 200 µl. After washing twice, each sample was incubated with its respective fluorophore-conjugated secondary antibody. Cells were then washed twice and resuspended in PBS. Stained cell samples were analyzed via flow cytometry using a FACSCanto II (BD Biosciences, San Jose, CA) and FlowJo software. Primary antibodies used were 8G5F11 (I1 in reference 14) and IE9F9 (I14 in reference 14) (Kerafast, Boston, MA), VSV-Poly, a kind gift from Hiroo Hoshino and Atsushi Oue (31, 32), and P5D4 (Sigma-Aldrich). Secondary antibodies used were Alexa Fluor 647-conjugated anti-mouse and anti-goat IgG (115-605-164 and 305-605-046, respectively; Jackson Immunoresearch, UK).

### Transient LV production and concentration.

Three-plasmid cotransfection into HEK293T cells was used to make pseudotyped LV as described previously (48). Briefly, 4 × 10^6^ 293T cells were seeded in 10-cm plates. Twenty-four hours later, they were transfected using FuGene6 (Promega, Madison, WI) with the following plasmids: SIN pHV (GFP expressing vector plasmid [50]), p8.91 (Gag-Pol expression plasmid [48]), and envelope expression plasmids. The medium was changed after 24 h, and then vector containing medium (VCM) was collected over 24-h periods for 2 days. Following collection, VCM was passed through Whatman Puradisc 0.45-µm filters (SLS) and concentrated ∼100-fold by ultracentrifugation at 22,000 rpm (87,119 × *g*) for 2 h at 4°C in a Beckmann Optima LK-90 ultracentrifuge using an SW-28 swinging-bucket rotor (radius, 16.1 cm). The virus was resuspended in cold plain Opti-MEM on ice, aliquoted, and stored at −80°C.

### LV titration.

The functional titer of each vector preparation was determined by flow cytometric analysis for GFP expression following transduction of HEK293T cells. Briefly, 293T cells (2 × 10^5^/well) were infected with LV plus 8 µg/ml Polybrene (Merck-Millipore, Billerica, MA) for 24 h. Infected cells were detected by GFP expression at 48 h following the start of transduction. Titers were calculated from virus dilutions where 1 to 20% of the cell population was GFP positive using the following formula:

$$\text{titer}(\text{TU}/\text{ml})=\left(\frac{\left(\text{no. of cells at transduction})\;\times (\%\text{GFP-positive cells}\;÷\;100)\times (\text{dilution factor}\right)}{\left(\text{volume of virus preparation added},\text{in ml}\right)}\right)$$

### Antibody neutralization assay.

To determine the neutralization activity of anti-VSVind.G monoclonal and polyclonal antibodies, an infection assay in the presence of antibodies was performed. Briefly, HEK293T cells were seeded in a 96-well plate at a density of 2 × 10^4^ cells/well with 200 µl of medium containing 8 µg/ml Polybrene. Approximately 3 h later, antibodies were serially diluted in plain Opti-MEM to 12 different concentrations/dilutions, ranging from 0.5 mg/ml (1:2 dilution) to 1.6 × 10^−7 ^mg/ml (1:6,250,000 dilution). Each antibody dilution was mixed 1:1 with VesG-LV or mutant G-LV at 4.0 × 10^5^ TU/ml to a final volume of 20 µl, incubated at 37°C for 1 h, and plated on the cells. Forty-eight hours later cells were harvested and analyzed for GFP expression by flow cytometry.

### SMD PCR for production of mutant G proteins for epitope mapping.

The site-directed mutagenesis (SMD) method was utilized to produce G protein mutants that were used in epitope mapping experiments. For this, a QuikChange II XL site-directed mutagenesis kit (Agilent, Santa Clara, CA) was used. Initially, primers that would have the desired nucleotide changes were designed using the QuikChange Primer Design Tool (http://www.genomics.agilent.com/primerDesignProgram.jsp). All primers used were obtained from Sigma-Aldrich (St. Louis, MO). The reaction was carried out according to manufacturer’s instructions.

### SG-PERT-based LV internalization assay and quantitative PCR assay.

A total of 2 × 10^4^ HEK293T cells/well were seeded in 24-well plates. Titers of 4.0 × 10^5^ TU/ml of VSVind.G- and RDpro-LV, as well as unenveloped LV (at a similar dilution), were mixed 1:1, vol/vol, with plain Opti-MEM or 0.1 mg/ml of 8G5F11 or IE9F9 to a total volume of 20 µl, incubated for 1 h at 37°C, and plated on cells. Following 30 min of incubation at 37°C, samples for SYBR green product-enhanced reverse transcriptase (SG-PERT) analysis (3 wells/condition) were harvested, washed, and treated with trypsin-EDTA (0.25%) (Gibco) for 30 min at 37°C. Cells then were lysed, and the SG-PERT was carried out as previously described (51, 52). In parallel, 5 h postincubation cells challenged with VSVind.G-LV were harvested (3 wells/condition), and total DNA was purified using the DNeasy Blood and Tissue kit (Qiagen, Germany). Fifty nanograms of DNA was subjected to SYBR green quantitative PCR using late RT (5′-CCCAACGAAGACAAGATCTGC-3′ and 5′-TCCCATCGCGATCTAATTCTCC-3′) and GFP (5′-CAACAGCCACAACGTCTATATCAT-3′ and 5′-ATGTTGTGGCGGATCTTGAAG-3′) primers to detect provirus as described previously (44). β-Actin (5′-TGGACTTCGAGCAAGAGATG-3′ and 5′-TTAAGTAGGCCGTCTTGCCT-3′) was used as the endogenous control. Infectivity was measured in parallel samples by flow cytometry 48 h postinfection.

### Statistical analyses.

All statistical analyses were performed using GraphPad Prism 5 software (GraphPad, La Jolla, CA). Details of all tests, including the calculated *P* values, are indicated in respective figure legends.
